# Evaluating the Impact of Virtual Reality on Orthopedic Trauma Skills Acquisition Among Surgical Residents: Randomized Crossover Study

**DOI:** 10.2196/79343

**Published:** 2026-05-06

**Authors:** Sandipan Chatterjee, Khairul Faizi Mohammad, Monica Ghidinelli

**Affiliations:** 1 AO Education Institute, AO Foundation, Clavadelerstrasse 8, Davos, 7270, Switzerland, 41 795725530; 2 Department of Orthopedics, Pantai Hospital Cheras, Kuala Lumpur, Malaysia

**Keywords:** virtual reality, orthopedic procedures, graduate medical education, simulation training, fracture fixation

## Abstract

**Background:**

Orthopedic trauma skills training is time-consuming and expensive. Current training modalities rely heavily on synthetic bone models, anatomical laboratory simulations, or assistance in surgeries (the apprenticeship model). Virtual reality (VR) appears to present a promising complement to current training modalities.

**Objective:**

This study evaluated the effectiveness of VR training on surgical performance and gauged learning preferences among orthopedic trauma residents in Malaysia.

**Methods:**

In total, 123 orthopedic residents were randomly assigned to 2 groups. One group practiced for about 30 minutes using VR glasses, followed by conventional nailing exercises on synthetic bones, while the other group first performed the nailing exercise, followed by VR practice. Performance was measured by time to completion of the exercise, and participants completed a postexercise survey.

**Results:**

Participants who completed VR training before the synthetic bone nailing exercise were significantly faster, completing the task between 4 (*P*=.05) and 7 (*P*=.002) minutes more quickly than without VR training. In addition, VR training improved self-assessed performance during the exercise. Survey data revealed that while 43% (50/117) of participants preferred conventional methods of learning (lectures, discussions, and hands-on simulations), 89% (104/117) of participants supported VR use as an adjunct to conventional methods of learning. Less than 2% (2/117, 1.7%) of participants indicated that conventional methods of learning were outdated.

**Conclusions:**

A single session of VR training significantly reduced completion times and improved self-assessment of competence in orthopedic trauma simulation exercises. Although learners continue to value conventional training modalities, there is a strong desire to include VR as a supplementary tool. Its integration into surgical curricula may accelerate skill acquisition, especially in low-resource settings with limited access to high-fidelity simulation labs. In addition, the availability of VR training modules in hospitals could help residents and junior consultants prepare for surgery.

## Introduction

### Background

The growing complexity of orthopedic trauma surgery has heightened the demand for advanced and effective training modalities. Conventional approaches such as synthetic bone models, anatomical laboratory simulations, and the apprenticeship model of learning through assisting in surgeries are time-consuming, expensive, and limited in availability [1,2]. Anatomical laboratory simulations provide high anatomical fidelity and tissue realism but are logistically demanding (eg, cadaver procurement, facility access, higher per-learner cost, and lower frequency). These constraints, particularly pronounced in low-resource settings, limit sufficient exposure to trauma case variations and present barriers to skill acquisition.

### Prior Work

Alternatives are being explored to complement traditional training modalities. Virtual reality (VR) has emerged as a promising tool that offers immersive, repeatable, and cost-effective simulations in a controlled environment [3-5]. In contrast to anatomical laboratories, immersive VR has standardized scenarios, providing immediate feedback and repetition at scale. VR also offers a lower marginal cost and greater scheduling flexibility, albeit with reduced tactile realism and haptic resistance compared with cadaveric tissue. Recent studies have highlighted the benefits of VR for orthopedic surgery, where procedural repetition and real-time feedback, which are critical for skill acquisition, can help reduce the learning curve [6]. VR in orthopedic training appears particularly effective in simulating procedures such as intramedullary nailing, arthroscopy, and arthroplasty [7-10]. Beyond technical skill acquisition, VR can also support the development of nontechnical skills, including decision-making and teamwork—critical elements in high-stakes surgical environments [11,12].

One of VR’s primary advantages is its scalability, particularly in low-resource settings [13]. The increasing availability of VR platforms such as Oculus Rift and HTC Vive has the potential to increase training opportunities in such regions [5]. The ability to simulate a broad range of clinical scenarios, including rare or complex cases, provides a comprehensive training experience that would otherwise be difficult to achieve through traditional methods.

Although prior studies have established VR’s general utility in surgical training, our study is the first to observe immediate skill transfer after a single VR session for both tibial and femoral nailing procedures, in a Malaysian residency context. Additionally, we examined the effect of VR training on self-assessed performance and gauged learner preferences for different training modalities.

### Training Challenges in Malaysia

In Malaysia, orthopedic residency is a 4-year training program under the Ministry of Health and the Ministry of Education. Upon graduation, residents serve in hospitals run by either the Ministry of Health or the Ministry of Education. Selection is based on open entrance examinations and interviews [14].

Surgical training in Malaysia is generally considered “in work training.” Hence, the level of training gained depends on the level provided by the trainer at the hospital in which the candidate is serving. The training level also varies depending on the opportunity to perform surgery and the types of cases available within a particular hospital. Other constraints particular to the region are the lack of sufficient hospital- or university-based courses. Anatomical laboratories are available but are expensive and infrequent [15].

According to a report published by the University of Malaya, there were an estimated 900 orthopedic surgeons in Malaysia in 2018, giving a ratio of 1 orthopedic surgeon per 40,000 population [14]. To meet the suggested World Health Organization ratio of 1:30,000 population, orthopedics in Malaysia faces the challenge of increasing surgeon numbers accordingly. Although approximately 70 residents are recruited annually and placed at 30 accredited training centers, they must also navigate economic realities to provide the best treatment within the limitations of public funding and patient affordability [14].

### Goal of This Study

This study aimed to evaluate the impact of VR-based training on surgical performance and to explore learning preferences of orthopedic trauma residents in Malaysia.

## Methods

### Educational Intervention

The educational intervention was integrated into a 3-day course that combined lectures, small group discussions, and hands-on practical exercises. Participants practiced a common tibia nailing procedure and a femoral nailing procedure on both VR headsets and synthetic bones. VR training was conducted using Oculus Rift S headsets (Meta Platforms Inc), running Johnson & Johnson MedTech simulation software. The headset recorded procedure completion time, procedural steps, and errors, where applicable.

**Figure 1.**
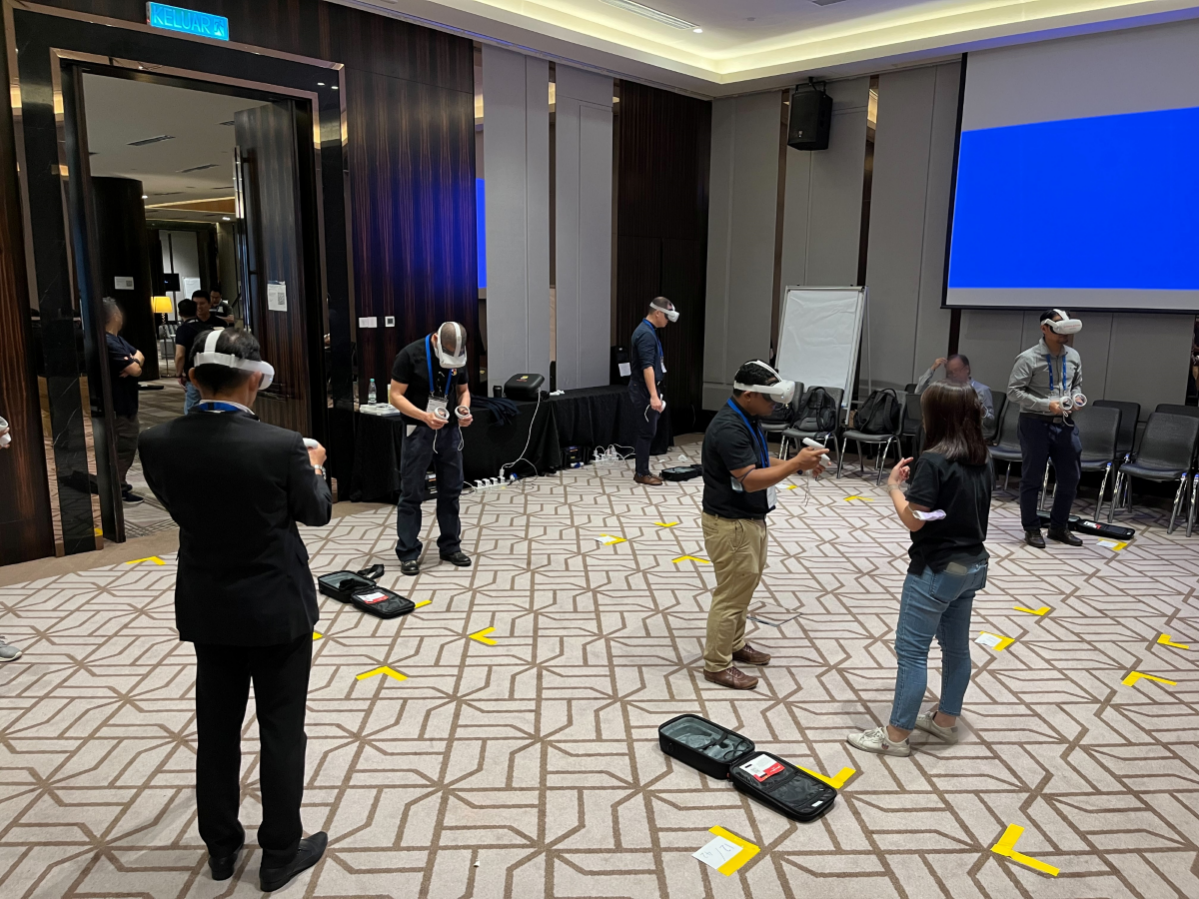
Setup of the virtual reality training session. Consent for using this image was obtained via course registration.

Each practical exercise workstation had 3 participants per synthetic bone. For VR training, participants practiced the nailing procedure once. In total, 30 VR headsets were available for this study, divided across 6 rooms, each with 5 participants, 1 faculty member, 1 laptop, and 1 technical staff member. The rooms were divided into individual grids of 1.5 m^2^ (Figure 1). The hotel Wi-Fi network was used to connect the headsets.

### Tibia Nailing Exercise

The tibia nail used for the synthetic bone exercise was the Expert Tibia Nail (Johnson & Johnson). For the VR module, the Tibia Nail Advanced (Johnson & Johnson) module was used. As the principal and major steps of both nailing systems are similar, they were deemed equivalent for comparison in this study. The tibia nailing procedure was performed during an AO (Arbeitsgemeinschaft für Osteosynthesefragen) Trauma Basic Principles course (cohort 1).

### Femoral Nailing Exercise

The femoral nail used for the synthetic bone exercise was the Trochanteric Fixation Nail Advanced Proximal Femoral Nailing System (Johnson & Johnson). The same nailing procedure was used for the VR module. The femoral nailing procedure was performed during the AO Trauma Basic Principles course (cohort 1) and the AO Trauma Advanced Principles course (cohort 2).

### Study Design

We used a 2-period crossover design. Participants were randomized to one of two sequences: (1) VR practice followed by the synthetic bone exercise or (2) the synthetic bone exercise followed by VR practice. After completing the first period, participants crossed over to the other modality. The primary end point was time (in minutes) to complete the synthetic bone exercise, with or without prior VR practice. These comparisons are reported separately for tibial and femoral procedures. Completion times for both modalities (VR and synthetic bone) are reported.

A qualitative postexercise survey assessed self-perceived performance and learning preferences. The questionnaire was developed, revised, and pilot-tested with 5 surgeons and administered via Microsoft Forms (version 16.0; Microsoft Corp).

### Population

Participants in this study were those who attended the AO Trauma Basic and Advanced Principles courses on the Principles of Fracture Management in Malaysia in March 2023. Beyond course attendance and consent, no inclusion or exclusion criteria were applied. All participants were able to complete VR training, as no cases of nausea or dizziness were observed while using the VR headsets.

### Data Collection

Participant demographic data were collected at baseline. For the synthetic bone exercises, each participant captured and reported the synthetic bone exercise completion time using a stopwatch. The completion times of the VR module were automatically captured via the VR headset software. Data were collected after each exercise and analyzed using Microsoft Excel (version 16.0; Microsoft Corp). Survey responses were collected after the exercise through Microsoft Forms.

### Analysis

A descriptive analysis was performed to determine the frequencies and percentages of the response selections. The tables and figures display frequency counts and/or percentages for each response option. A paired 2-tailed *t* test was used to test the effect of VR training on time to completion of the synthetic bone practical exercise. A Bonferroni post hoc test was conducted to correct for multiple testing.

### Ethical Considerations

According to the ethics committee of the Canton of Zurich, this study did not require ethical committee authorization (Req-2025-00605). Informed consent was obtained from participants prior to the start of the course and no compensation was provided to study participants for taking part in this study. In the survey, we included the following statement of purpose, which disclosed our intended use of the data: “The information you provide will be anonymized and made available to the study group in aggregate form. The data will be used for research purposes.”

## Results

### Participant Demographics

This study included 2 cohorts with 63 and 60 participants from the AO Trauma Basic and Advanced Principles courses, respectively (Table 1). The average age between these two cohorts was significantly different (*P*<.001), as was the average years of orthopedic trauma training (*P*<.001) (Table 1). There was no significant difference in the number of femoral nailing cases performed by the participants of each cohort.

**Table 1.** Participant demographics and experience level.^a^

Participant demographics		Cohort 1^b^	Cohort 2^c^	*P* value
Sex, n (%)				—^d^
Female		15 (24)	5 (8)	
Male		48 (76)	55 (92)	
Age (years), mean (SD)		33.0 (3.6)	37.36 (2.8)	<.*001*
Career stage, n (%)				—^d^
Specialist		14 (22)	57 (95)	
Master’s trainee		9 (14)	2 (3)	
Service medical officer (resident)		40 (64)	1 (2)	
Experience, mean (SD)				<.*001*
Duration of orthopedic training (years)		4.5 (3.2)	9.6 (3.3)	
Number of tibia nailing cases performed as a primary surgeon		10 (12)	—	
Number of tibia nailing cases performed as an assistant surgeon		15 (19)	—	
Number of femoral nailing cases performed as a primary surgeon		10 (13)	15 (20)	
Number of femoral nailing cases performed as an assistant surgeon		13 (16)	16 (24)	

a Averages include SDs, and *P* values when significant are italicized.

b Cohort 1: n=63.

c Cohort 2: n=60.

d Not applicable.

### Completion Time for Tibia Nailing Exercise

For each procedure, we compared the synthetic bone completion time among participants who performed VR practice before the synthetic bone task versus those who performed it after. For cohort 1, the survey response rates (proportion of participants who completed the postexercise survey) varied from 80% (50/63) to 92% (58/63). For cohort 2, the response rate was approximately 93% (55/60).

For the tibia nailing exercise, participants who trained on the VR module prior to performing the synthetic bone practical exercise were, on average, 5 minutes faster in completing the nailing exercise (P=.007; Table 2). A significant decrease in time needed to finish the VR module was also noted when participants first completed the synthetic bone practical exercise (*P*=.02; Table 2).

**Table 2.** Completion times for the tibia nailing exercise.

Cohort 1	VR^a^ module then synthetic bone exercise, mean (95% CI)	Synthetic bone exercise then VR module, mean (95% CI)	*P*^*b*^ *value*
Time (min) to complete synthetic bone exercise	18.48 (16.19-20.77)	23.43 (20.8-26.01)	*.007*
Time (min) to complete VR module	34.97 (31.51-38.44)	29.82 (27.43-32.2)	*.02*

a VR: virtual reality.

b Italicized *P* values are significant.

### Completion Time for Femoral Nailing Exercise

Participants from cohort 1 who trained on the VR module prior to performing the synthetic bone practical exercise were, on average, approximately 7 minutes faster in completing the femoral nailing exercise (*P*<.01; Table 3). No significant difference in the VR module completion time was noted after participants performed the synthetic bone exercise (*P*=.09; Table 3). Similarly, in cohort 2, participants were on average approximately 4 minutes faster in completing the synthetic bone practical exercise after training on the VR module (*P*=.05; Table 3), and no significant difference in the VR module completion time was noted (*P*=.54; Table 3).

**Table 3.** Completion times for the femoral nailing exercise.

Cohort and outcome	VR^a^ module then synthetic bone exercise, mean (95% CI)	Synthetic bone exercise then VR module, mean (95% CI)	*P^b^* value
Cohort 1			
Time (min) to complete synthetic bone exercise	20.71 (18.11-23.32)	27.55 (24.25-30.86)	*<*.*01*
Time (min) to complete VR module	18.65 (16.82-20.48)	16.69 (15.36-18.01)	.09
Cohort 2			
Time (min) to complete synthetic bone exercise	16.51 (15.03-17.98)	20.73 (16.89-24.58)	.*05*
Time (min) to complete VR module	21.57 (19.65-23.49)	22.45 (20.42-24.48)	.54

a VR: virtual reality.

b Italicized *P* values are significant.

### Impact of VR Training on Self-Assessed Performance

Analysis of the qualitative questionnaire showed that after VR training, a higher number of participants rated the ease of understanding the nailing practical exercise in the “extremely easy” and/or “very easy” categories. This trend (results not statistically significant) was also noticed when participants were asked to evaluate their performance during the synthetic bone exercise (with the exception of cohort 1 for femoral nailing). This observation was seen for both the tibial and femoral nailing exercises (Table 4 and Table 5).

**Table 4.** Impact of virtual reality (VR) training on understanding and performance during the tibia synthetic bone nailing exercise.

Tibia nailing outcomes	Synthetic bone exercise without VR training, n (%)	Synthetic bone exercise with VR training, n (%)
How would you rate the ease of understanding the practical?		
Extremely easy	0 (0)^a^	4 (13)^b^
Very easy	9 (30)^c^	10 (33)^d^
Easy	17 (57)^d^	10 (33)^d^
Somewhat easy	4 (13)^b^	6 (20)^c^
Not at all easy	0 (0)^a^	0^a^ (0)
How would you rate your performance in this practical?		
Excellent	2 (7)^b^	4 (13)^b^
Very good	9 (30)^c^	13 (43)^d^
Good	19 (63)^d^	13 (43)^d^
Poor	0^a^ (0)	0^a^ (0)
Very poor	0^a^ (0)	0^a^ (0)

a Lowest frequency of responses.

b Moderate frequency of responses.

c High frequency of responses.

d Highest frequency of responses.

**Table 5.** Impact of prior virtual reality (VR) training on understanding and performance during the femoral synthetic bone nailing exercise.

Femoral nailing outcomes	Cohort 1		Cohort 2	
	Synthetic bone without VR training, n (%)	Synthetic bone with VR training, n (%)	Synthetic bone without VR training, n (%)	Synthetic bone with VR training, n (%)
How would you rate the ease of understanding the practical?				
Extremely easy	0 (0)^a^	4 (13)^b^	1 (3)^a^	4 (15)^b^
Very easy	3 (10)^c^	5 (16)^b^	8 (29)^c^	9 (33)^c^
Easy	21 (73)^d^	13 (42)^d^	13 (46)^d^	13 (48)^d^
Somewhat easy	3 (10)^c^	8 (26)^c^	5 (18)^b^	1 (4)^a^
Not at all easy	2^b^ (7)	1 (3)^a^	1 (3)^a^	0 (0)^a^
How would you rate your performance in this practical?				
Excellent	0^a^ (0)	2 (7)^b^	5 (18)^c^	4 (15)^b^
Very good	10 (35)^c^	7 (23)^c^	3 (11)^b^	10 (37)^c^
Good	16 (55)^d^	19 (61)^d^	18 (64)^d^	12 (44)^d^
Poor	3 (10)^b^	2 (7)^b^	2 (7)^b^	1 (4)^b^
Very poor	0^a^ (0)	1 (3)^a^	0 (0)^a^	0 (0)^a^

a Lowest frequency of responses.

b Moderate frequency of responses.

c High frequency of responses.

d Highest frequency of responses.

### Learning Preferences

When asked to compare VR with conventional methods of learning (lectures and synthetic bone practical exercises), participants were strongly in favor of VR as a supplement to conventional methods of learning. Only 3.4% (4/117) chose VR modules alone as their preferred method of learning, 89% (104/117) preferred VR as an adjunct to conventional methods, and only 8% (9/117) believed VR added no value (Table 6).

When asked about the reverse, evaluating conventional methods of learning versus VR, 43% (50/117) preferred conventional methods of learning, 56% (65/117) preferred conventional methods as an adjunct to VR, and less than 2% (2/117, 1.7%) considered conventional methods to be outdated (Table 6).

**Table 6.** Participant learning preferences.

Learning preference	Participants, n (%)
How do you rate the VR^a^ module vs conventional methods of learning (lectures and hands-on training with synthetic bones)?	
Prefer the VR module	4 (3.42)
Prefer VR as an adjunct to conventional methods of learning	104 (88.89)
VR does not add to conventional methods of learning	9 (7.69)
How do you rate conventional methods of learning (lectures and hands-on training with synthetic bones) vs the VR module?	
Prefer conventional methods of learning	50 (42.74)
Prefer conventional methods of learning as an adjunct to VR teaching	65 (55.56)
Conventional methods of learning are outdated	2 (1.71)

a VR: virtual reality.

### Association of Age and Experience Level With Completion Times of the Femoral Nailing Exercise and VR Module

As the average age of the 2 cohorts were significantly different (33.0, SD 3.6 years for cohort 1 and 37.6, SD 2.8 years for cohort 2; *P*<.001; Table 1) and the average number of years of orthopedic training was also significantly different (4.5, SD 3.2 years vs 9.6, SD 3.3 years; *P*<.001; Table 1), we conducted an unplanned subanalysis examining the influence of age and experience on completion times of the femoral nailing exercise. Participants in cohort 2 (older, with more years of training) were significantly faster in completing the synthetic bone nailing exercise than those in cohort 1 (20.7 vs 27.5 minutes; *P*=.01; Table 7). However, when comparing the VR module completion times, cohort 1 (younger, with fewer years of training) was significantly faster than cohort 2 (18.6 vs 21.8 minutes; *P*=.03; Table 7).

**Table 7.** Effect of age and experience on completion time of nailing exercises.

Outcomes	Cohort 1 (time in minutes)	Cohort 2 (time in minutes)	*P^a^* value
Time to complete the femoral nailing *synthetic bone* exercise (without virtual reality training)	27.5	20.73	*.01*
Time to complete the femoral nailing *virtual reality module* (without prior synthetic bone exercise)	18.65	21.57	*.03*

a Italicized *P* values are significant.

## Discussion

### Principal Results

This study demonstrated immediate skill transfer from a single VR session to synthetic bone performance across 2 orthopedic procedures in a Malaysian residency context.

Our primary finding suggests that VR supports rapid skill acquisition and transfer to synthetic bone models. Additionally, we explored learning preferences, which could inform future curriculum design in low-resource countries. Although traditional hands-on training remains highly valued, most participants viewed VR positively as a tool complementary to conventional training methods. Our qualitative data further support VR’s effectiveness, with participants reporting a better understanding of the practical exercises and higher self-rated performance after VR training.

An interesting secondary finding emerged from our unplanned subanalysis comparing cohorts of different experience levels. The more experienced participants performed the conventional synthetic bone femoral nailing exercise significantly faster than the less-experienced group. This result confirms that conventional surgical skills improve with years of practice.

In contrast, the younger and less-experienced cohort completed the VR module significantly faster. This finding suggests that younger trainees may adapt more quickly to digital learning environments, possibly due to greater familiarity with technology. However, given baseline differences and the pragmatic study design, the analysis of age and experience is exploratory and should not be interpreted as causal.

Several practical implications emerge from our findings. First, in the context of Malaysia’s orthopedic training landscape, where there is a need to train more surgeons to meet the World Health Organization–recommended ratio of 1:30,000 population, VR offers a scalable supplement that could help standardize training while optimizing the use of limited resources. With limited access to cadaveric specimens and economic constraints, VR platforms could provide residents with additional repetitions of procedures before they enter the operating room.

Second, the demonstrated reduction in time to complete practical exercises after VR training shows the transferability of skills from VR to real-life settings. Incorporating VR into preprocedural preparation could improve operating room efficiency. This is particularly valuable in resource-constrained settings where operating room time is limited and maximizing throughput is essential.

Third, the improved self-assessment of performance after VR training indicates enhanced procedural confidence, which could translate to reduced stress and potentially better outcomes in actual surgical situations. The combination of faster performance and increased understanding suggests that VR training may help flatten the learning curve for complex surgical procedures and offers a solution that complements rather than replaces traditional training methods [16,17].

### Comparison With Prior Work

The growing body of evidence on the use of VR simulations for orthopedic surgery skills training and education has resulted in VR being included as part of the third technological wave in orthopedics [18]. Although there are still some questions concerning cost-effectiveness and skill retention using VR simulations, there is no doubt about VR’s effectiveness in surgical training, particularly in orthopedics, where procedural repetition and spatial understanding are critical [19,20]. Our work aligns well with this literature, as we demonstrate immediate skill transfer after a single VR training intervention for not 1 but 2 orthopedic procedures.

A strong preference for a mixed-learning approach (conventional and VR) among participants reflects the need to rethink the role of VR in surgical skills training, especially in the context of low-resource settings [21,22], and to offer a scalable solution that complements rather than replaces traditional training methods. Acquiring VR headsets would be a 1-time investment for the training facility or could be sourced from companies willing to sponsor the event. On the other hand, skills training on synthetic bones requires the purchase of bone models for every participant and training event.

### Strengths

This study benefits from several methodological strengths. The crossover design allowed each participant to experience both training modalities, reducing individual variation as a confounding factor. The relatively large sample size (N=123) and the inclusion of 2 distinct cohorts with different experience levels enhance the generalizability of our findings. Additionally, testing 2 different nailing procedures (tibia and femoral) demonstrates that the observed effects are not procedure specific.

### Limitations

Several limitations warrant consideration when interpreting our results. First, completion times for the synthetic bone exercises were self-reported, whereas VR module completion times were captured by the software. This could introduce a reporting bias between the 2 modalities. Although we provided participants with standardized instructions, reporting errors cannot be excluded. Future studies could benefit from objective timing methods or video recording of procedures for more accurate measurement. Second, we evaluated effectiveness primarily through completion time, which, while important, represents only 1 dimension of surgical competence. Additional outcome measures such as procedural accuracy, error rates, or expert assessment of technique would provide a more comprehensive evaluation of VR’s impact on skill acquisition. Third, our study assessed immediate skill transfer but did not include follow-up assessments to determine the durability of learned skills. Longitudinal studies would be valuable to assess whether VR-acquired skills persist over time and translate to improved performance in actual surgical cases. Finally, this was a pragmatic, course-embedded evaluation. Beyond course attendance and consent, we did not apply formal inclusion or exclusion criteria, which may contribute to baseline heterogeneity. Using immersive VR headsets has been shown to cause dizziness or nausea (cybersickness) [23]. Although we did not experience any such cases in this study, it needs to be considered while planning similar interventions.

### Conclusions

This study provides new evidence that even a single, brief VR training session can significantly decrease completion times of simulated nailing procedures. This observation, coupled with improved self-reported procedural understanding and performance, suggests that VR represents a valuable educational tool for orthopedic skills training.

Although conventional hands-on training remains an integral part of residency training, there is a strong preference for a mixed approach incorporating VR technologies. In the context of Malaysia’s need to train more orthopedic surgeons, VR training could offer a scalable complement to traditional methods that could help standardize training while optimizing resource use. This study also highlights the need for tailored implementation strategies that account for varying levels of technological familiarity. Finally, as VR technology continues to evolve and become more accessible, its integration into orthopedic training curricula represents a promising approach to enhance surgical education.

## Abbreviations

AO: Arbeitsgemeinschaft für Osteosynthesefragen
VR: virtual reality
